# Physical and chemical characterization of smokeless tobacco products in India

**DOI:** 10.1038/s41598-023-35455-3

**Published:** 2023-06-01

**Authors:** Priyamvada Sharma, Nuan Ping Cheah, Jagdish Kaur, Sandhya Sathiya Kumar, Vijayashree Rao, Faridatul Akmam Morsed, Michelle Yong Bing Choo, Pratima Murthy

**Affiliations:** 1 Department of Clinical Psychopharmacology and Neurotoxicology and Department of Psychiatry, National Institute of Mental Health and Neurosciences (NIMHNAS), Bangalore, India; 2 Present Address: Director Cigarette Testing Laboratory, Applied Sciences Group, Health Sciences Authority, 11 Outram Road, Singapore, Singapore; 3 Regional Adviser (Tobacco Free Initiative) Department of Healthier populations and Noncommunicable Diseases, WHO Regional Office for South-East Asia, World Health House, I.P. Estate, M.G. Road, New Delhi, India; 4 Toxicology Laboratory, Centre for Addiction Medicine, Department of Psychiatry, National Institute of Mental Health and Neurosciences (NIMHNAS), Bangalore, India; 5 Cigarette Testing Laboratory, Pharmaceutical Division, Applied Science Group, Health Sciences Authority, 11 Outram Road, Singapore, 169078 Singapore; 6 Director and Senior Professor of Psychiatry, National Institute of Mental Health and Neurosciences (NIMHANS), Bangalore, 560029 India

**Keywords:** Chemical biology, Health care, Medical research, Chemistry

## Abstract

The rapid proliferation of smokeless tobacco (SLT) in India has occurred without adequate information on the possible dangers and toxicity of these products. Tobacco flavors as well as nicotine (both protonated and un-protonated) are responsible for health dangers and addiction. The study aimed to offer information on the physical characteristics of commonly used smokeless tobacco products (including microscopic analysis), along with nicotine content (both total and un-protonated), pH, moisture, and flavors. The Standard Operating Procedures (SOPs) validated by the World Health Organization (WHO) recognized Tobacco Testing Laboratory TobLabNet) were applied for the analysis of various constituents of the SLTs. The microscopic analysis indicated that some of the SLT products like khaini were finely processed and available in filter pouches for users’ convenience and prolonged use leading to prolonged retention and addiction potential. Nicotine absorption and availability (both protonated and un-protonated) are affected by moisture and pH. Essences provide a pleasant aroma and flavor, with an increased risk of misuse and other health problems. Few chewing tobacco and Zarda had the lowest levels of un-protonated nicotine (0.10–0.52% and 0.15–0.21%, respectively), whereas Gul, Gudhaku, and Khaini had the highest levels, ranging from 95.33 to 99.12%. Moisture and pH ranged from 4.54 to 50.19% and 5.25–10.07 respectively. Menthol (630.74–9681.42 µg/g) was the most popular flavour, followed by Eucalyptol (118.16–247.77 µg/g) and camphor (148.67 and 219.317 µg/g). SLT’s health concerns and addiction dangers are exacerbated by the high proportion of bioavailable nicotine coupled with flavors. The findings of this study have important implications for the regulation and use of SLT in countries where use of SLT is prevalent.

## Introduction

Smokeless tobacco is a complex chemical mixture that contains a variety of chemicals and additives, including flavors, areca nut, and slaked lime, and used with betel leaves^1^. Smokeless Tobacco (SLT) products are extremely complex, containing almost 4000 compounds, many of which are hazardous, mutagenic, and carcinogenic^2^ in nature. The alkaloid nicotine, the primary addictive substance in tobacco^3–5^, exists in protonated and un-protonated forms^6^. The addition of slaked lime in the preparation of SLT enhances nicotine bioavailability^7,8^.

Betel quid with tobacco, Khaini, Gutka, Pan Masala with tobacco, Zarda, Mishri, Mawa, Gul, Bajjar, Gudhaku, and other SLT products are widely available and used in India. These items can be chewed, sucked, or placed between the cheek, gum, or teeth^9,10^. Bangladesh, Bhutan, India, Myanmar, Nepal, Sri Lanka, and Timor-Leste are among the countries in the South-East Asian Region (SEAR) with the highest prevalence of SLT use^11^. Prevalence of current SLT use among men is highest in Myanmar (62.2%), and among women in Timor-Leste (26.8%)^11^. As per a recent study, during 2015–2019, there were 165 803 900 SLT users across SEAR, with 479 466 attributable deaths annually, of which India accounted for 79.9%, with 383 248 deaths.

According to the Global Adult Tobacco Survey-2, (GATS 2), every third adult in rural India and every fifth adult in urban India consumes tobacco in some form or another. Thus, 28.6% (266.8 million) of adults in India aged 15 and above use tobacco in some form. The prevalence of tobacco usage in India is 42.4 percent among men and 14.2 percent among women^12^. The most popular tobacco product is Khaini (tobacco and lime mixture), which is used by one in every nine adults (11.2%), followed by bidi, which is smoked by 7.7% of adult Indians^11^ Gutkha (a mixture of tobacco, lime, and areca nut) is ranked third (6.8%), and betel quid with tobacco is ranked fourth (5.8%). In India, 18.4% of women use SLT, and because smoking is typically a socially taboo (GATS 2), SLT is used as an alternate and more acceptable form of tobacco intake^12,13^. Affordability and accessibility lead to increased use of smokeless tobacco products, including through illicit trade. Even in instances of jurisdictional ban, the sale and possession of smokeless tobacco products, continue through illicit means (10).

The vast majority of SLT products are typically blended with herbs, spices, areca nuts, betel leaves, and slaked lime and made in the unorganized sector, where they are poorly regulated^14^ and contain significant quantities of tobacco, increasing the possibility of abuse and long-term dependence^15–17^. Nicotine is a basic alkaloid that remains un-ionized at an alkaline pH and is responsible for tobacco addiction. Nicotine absorption is influenced by several factors, including concentration, moisture content, flavorings, and pH. The pH of the product influences nicotine absorption; a higher pH accelerates the production of free-base nicotine (the most potent and easily absorbed form of nicotine), resulting in better absorption via the oral mucosa. As a result, pH information, in addition to nicotine, is a crucial indicator of bioavailable free nicotine. Moisture content affects nicotine absorption; a product with a higher moisture content absorbs more nicotine than one with a lower moisture content^18–20^.

Due to its distinctive scent, taste, and appeal, flavor additives are an essential aspect of SLT products^21,22^. Mint, spearmint, and wintergreen have been around for a long time, and menthol is used to soften the harshness of tobacco^23,24^ and make it more appealing to young people and beginners^25^. The health dangers of SLT are unknown because they have not been thoroughly explored, and professionals do not have much evidence on which to base their opinions. The aim of this study was to investigate the chemical composition and microscopic examination of frequently used SLT products in India in order to produce evidence regarding the potential health risks, hazards, and toxicity of these products. Microscopical analysis and signature data on the chemical components of well-known products are both necessary to ascertain the physical characteristics and quality of tobacco used. Microscopic analysis is considered a traditional, rapid, well-approved, and cost-effective approach for the identification of plant products. Since SLT products contain various species/ flavors and ingredients, it helps to identify products containing tobacco. Thus, simple microscopic techniques were used to identify the tobacco ingredients in SLT products. For the examination of the chemical contents of SLT products, gas chromatography (GC) with various detectors (Flame Ionization, Mass detector) is favored and is the suggested standard approach^26,27^. In this research we describe an easy-to-use and quick analytical process for determining nicotine utilizing gas chromatography-flame ionization detection, developed by WHO TobLabNet 12^28^. For the chemical ingredient analysis, GC–MS-based techniques were applied to identify, list and quantify the possible flavors added to the SLT products^18,28^.

## Material and methods

All chemicals used for analysis were of analytical grade. Solvents and routine chemicals were procured from SISCO research laboratory (Mumbai, India). Nicotine, Quinoline (Internal standard), flavors (Eucalyptol, Camphor, Menthol, Methyl Salicylate, Ethyl salicylate, Cinnamaldehyde, Eugenol, Diphenyl ether, and Coumarin) and its internal standard (3′, 4′-(Methylenedioxy) acetophenone—MDA) were procured from Sigma Aldrich, USA having purity of ≥ 99.0%).

### Patient and public involvement

Patients were not involved in this research.

### Samples

A total of twenty-one brands of SLT and Pan masala were randomly sourced from retail shops or vendors selling tobacco products in India's north, east, west, and central regions for this study. The sample included one brand each sample of Khaini, Gudhaku, and Kharra, two of Zarda and Mawa, six of chewing tobacco, three of Gul, and five of Pan Masala. As per IARC, International Agency for Research on Cancer, Pan Masala is a ready-to-eat commercially available sachet containing areca nut crushed into very small pieces, slaked lime, catechu, and condiments with or without powdered tobacco). The samples were transported in airtight sealed packs, to the Drug Toxicology Laboratory, Centre for Addiction Medicine (CAM), National Institute of Mental Health and Neurosciences (NIMHANS), Bengaluru, India. The samples were stored in plastic bags and stored in a Thermo Scientific Ultra Low deep freezer at – 20 °C. Prior to analysis, samples were refrigerated for 24 h for comprehensive re-equilibration, followed by 2 h of equilibration to ambient conditions. The University of Kentucky’s College of Agriculture provided Coresta Reference Tobacco Products: CRP1.1, CRP2.1, CRP3.1, and CRP4.1 as reference material for method validation. Ethics clearance for method standardization and quantification of commonly abused substances was obtained.

SLT products and Pan Masala used in this study are classified and tabulated based on the way in which they are consumed by the user (chewing, sucking by placing between gums and cheek for a gradual release of the ingredients and applied on teeth and gums as dentifrice) tabulated in Table 1.

**Table 1:** Types and mode of ingestion of Smokeless Tobacco Products.

Sucking	Chewing	Applied on teeth and gums
Kharra	Tobacco (for chewing)	Gudakhu
Khaini	Kharra	Gul
Loose Tobacco	Mawa	
	Zarda	
	Pan Masala	

### pH, moisture and nicotine analysis

The state-of-the-art Drug Toxicology Laboratory at NIMHANS, Bangalore, is a world-class facility equipped with high-end and sophisticated instruments for tobacco analysis, as well as a member of the World Health Organization (WHO) commissioned Tobacco Laboratory Network (TobLabNet). TobLabNet aims to validate analytical approaches and Standard Operating Procedures (SOP) for evaluating smokeless tobacco constituents and smoked tobacco emissions across the globe.

Moisture and pH were evaluated using (TobLabNet) SOP 13 and 14 on a Thermo Scientific Heratherm oven QM5180 and an Orion Star A211 pH meter. Nicotine quantification was performed in accordance with SOP12, which permitted the use of FID detector in conjunction with a 7890A gas chromatography. The flavors were quantified using Agilent Technologies' 5975C mass selective detectors (GC MSD)^29^.

Nicotine stock standard solution (2 g/L) was made by dissolving nicotine in a 2:1:4:1 ratio of water, extraction solution, and 2 M sodium hydroxide. Internal standard, n-heptadecane diluted in n-hexane, was included in the extraction solution (0.5 mg/ml). To mix the nicotine stock standard solution, it was shaken for roughly 60 min in an orbital shaker. Following phase separation, the supernatant organic solution was used to make nicotine working standards in concentrations of 50, 250, 500, 750, 1000 and 1500 mg/L, which were serially diluted with the extraction solution. The produced solutions were kept at 4–8 °C and shielded from light.

### Flavor analysis

Nine different flavors (Eucalyptol, Camphor, Menthol, Methyl salicylate, Ethyl salicylate, Cinnamaldehyde, Eugenol, Diphenyl ether, and Coumarin) were analyzed according to the work done by Stanfill SB, 2018 (29, 30) and CDC TL-Method 060.

### Microscopic examination

The microscopic images were captured on a Leica digital microscope DM6 B, Germany and Leica digital microscope DVM 6, Singapore on Leica LAS X 3.0.8, Microsystems CMS GmbH. A Leica digital microscope DM6 B was used to undertake microscopic cellular examinations^30,31^. A small amount of the sample was combined with enough water to make a fragment, which was then extracted for microscopic analysis. Samples were inspected directly using a Leica digital microscope DVM 6 for 3-D imaging.

### Ethics approval and consent to participate

This work does not involve human samples and hence ethics exemption for this particular study was obtained.

## Results

**Figure 1:**
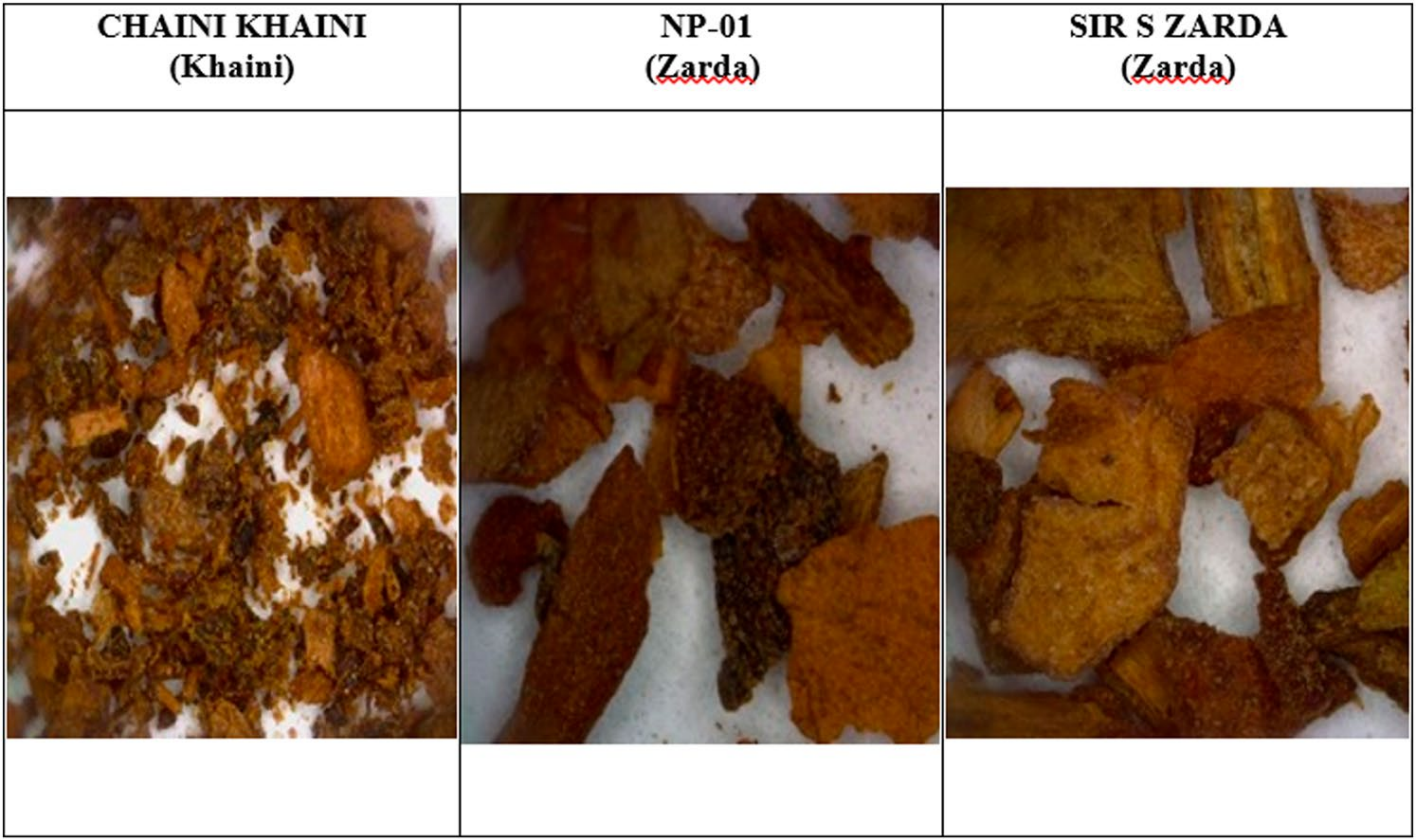
3-D Images of smokeless tobacco products (SLT).

**Figure 2:**
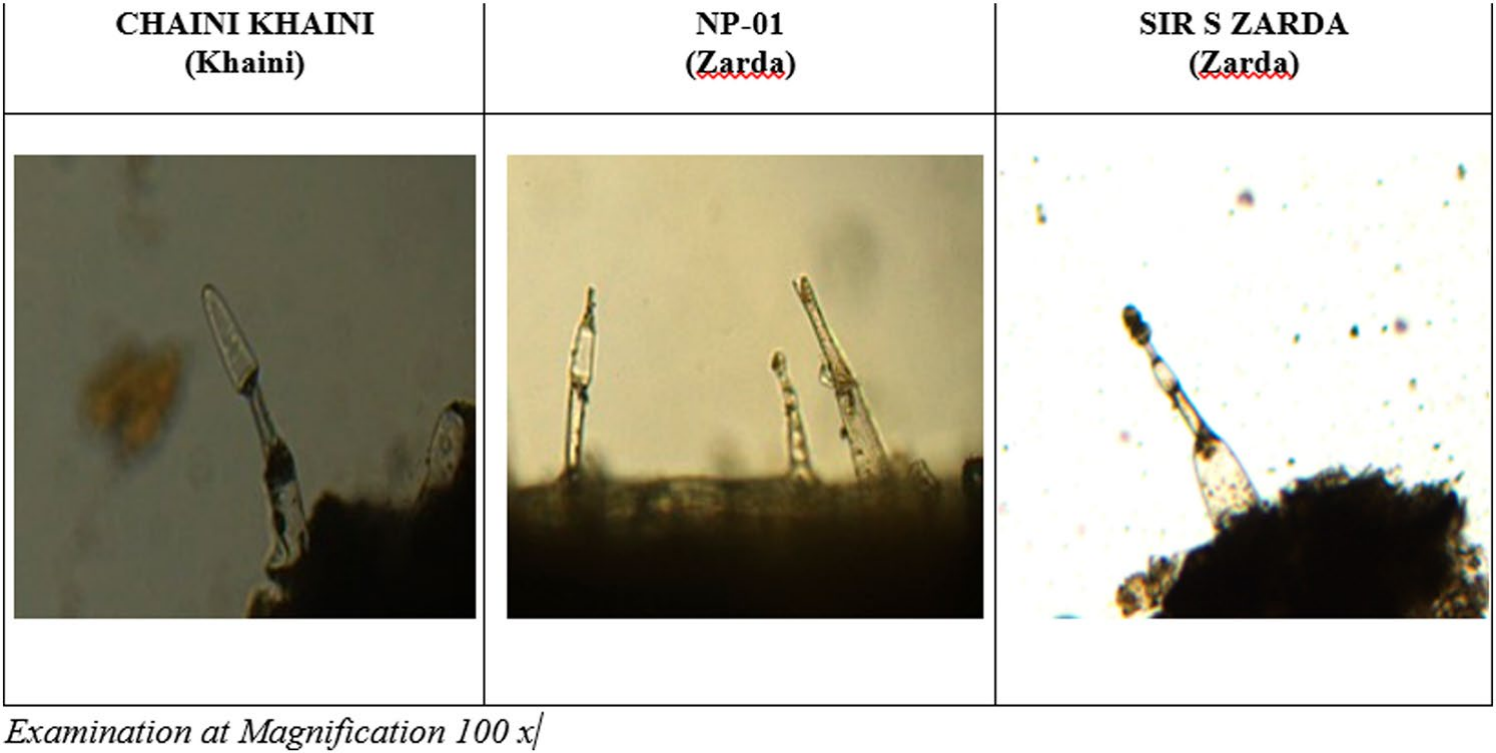
Cellular Microscopic Images of Tobacco Trichrome for 3 smokeless tobacco products.

Simple microscopic analysis focused on identifying the unique characteristics of the tobacco plant. Trichomes were found to be unicellular or multicellular epidermal appendages (Figs. 1, 2) with distinct morphologies (31). Multicellular glandular trichomes resemble epidermis outgrowths with a head of cells (Fig. 2) and secrete or store significant amounts of particular metabolites for plant's chemical defense mechanism^30^. Microscopic examination confirmed the presence of tobacco in SLT, which also includes a number of spices and flavors.

Similarly, control samples, Chaini Khaini reference product, and CM 9 (Coresta Monitor Cigarette) were studied under comparable conditions and revealed Chaini Khaini was finely processed as compared to Zarda samples (NP-01 and Sir S Zarda) in a 3-D inspection with Leica digital microscope DVM 6 to acquire an in-depth study on the physical content of the products. The Chaini Khaini 3-D capture revealed nicely cut material, however the Zarda items were coarsely cut and sized irregularly (Fig. 1).

The Chaini Khaini appeared flakier than the reference material, implying that the product has been thoroughly processed or that other ingredients have been added. The pH of Khaini, on the other hand, is in the alkaline range (pH 8–10).

The results of nicotine, un-protonated nicotine, pH, and moisture of the SLT products studied are presented in Table 2.

**Table 2:** Mean of moisture, pH, nicotine (total and unprotonated μg/gm) measured in triplicate in Smokeless Tobacco Products.

Smokeless tobacco type	Name of the product	Moisture content %	pH	*Nicotine content mg/g	*Un-protonated nicotine content mg/g	% Un-protonated nicotine
Gudhaku (Tobacco paste-like preparation)	Tota chaap	15.48	9.62	5.81	5.67	97.55
Kharra (Mixture of areca nut and tobacco)	Golden babu	8.49	8.17	7.37	4.31	58.55
Khaini (Sun-dried/fermented tobacco leaves)	Chaini khaini	22.78	9.33	4.67	4.45	95.33
Gul (Oral tobacco powder)	AR chand gul	8.64	8.59	28.23	22.24	78.79
	Jora panja gul	4.54	10.07	22.02	21.82	99.12
	Gulab marka Gul	5.98	9.63	31.25	30.50	97.60
Zarda (Moist/dry chewing tobacco with spice essences, and perfumes)	NP-01	11.61	5.20	21.72	0.03	0.15
	Sir S zarda	12.24	5.35	17.91	0.04	0.21
Chewing tobacco (Cured tobacco leaves)	No brand- Loose tobacco	17.73	8.58	17.26	13.53	78.41
	P P tobacco	14.78	5.41	17.24	0.04	0.24
	Kuber tobacco	50.19	8.42	6.99	5.00	71.53
	Madhu chewing tobacco	8.76	5.74	14.28	0.07	0.52
	BHR chewing tobacco	13.74	5.25	26.13	0.03	0.10
	Double black royal touch	12.1	5.43	23.08	0.06	0.26
Pan masala (Mixture of areca nut with slaked lime, catechu and other flavoring agents with or witout tobacco.)	Kamala pasand pan masala	8.19	8.90	0.00	0.00	88.35
	Pan parag premium	5.67	8.79	0.00	0.00	85.48
	Rajnigandha pan masala	7.47	8.97	0.00	0.00	89.91
	Vimal pan masala	5.37	8.83	0.00	0.00	86.59
	Raj niwas premium	6.26	8.70	0.00	0.00	82.72

*Dry weight.

The moisture content among the SLT products (n = 19) ranged from 4.54 to 50.19%. The average moisture percentage was 12.63 ± 10.28. The coefficient of variation was 83.96%, indicating wide dispersion across brands. Kuber tobacco had the highest moisture content (50.19%), while Gul of the brand Jora Panja had the lowest moisture content (4.54%). For the Coresta standards, moisture contents were within the mentioned range (Table 3). Gudakhu, Kharra, and Khaini have low to high moisture content. We found that moisture content was inversely related to nicotine, particularly Gul products, where unprotonated nicotine was 78.79–99.12% and moisture was 4.43–8.64%. pH is another major component that affects nicotine pharmacokinetics. As pH rises, the fraction of unprotonated nicotine increases and is easily absorbed through the buccal mucosa. Altering the pH of a product can greatly increase nicotine absorption and influence its abuse potential. The pH of the products analyzed ranged from 5.25 to 10.07, the average was 7.84 ± 1.77 the coefficient of variation was 22.52%. pH for Coresta standards was 6.08–8.30, well within the range (Table 3).

**Table 3:** Moisture, pH and nicotine analysis in Coresta reference standards.

Name of the product	Moisture content %	pH	Nicotine content (mg/g)
CRP1.1	54.64	7.42	7.07
CRP2.1	51.10	7.94	9.75
CRP3.1	5.78	6.99	16.39
CRP4.1	22.29	5.89	9.24

The nicotine concentrations in SLT products ranged from 4.67 to 28.23 mg/g (Table 1), average was 12.84 and the coefficient of variation was 83.96%, The unprotonated nicotine was calculated by putting the product pH and appropriate Pka into the Henderson-Haselbalch equation^29^ it ranged from 0.10 to 99.1% and the coefficient of variation was 71.38%, indicating great heterogeneity across brands. Products with an acidic pH (5.20–5.74) had 0.12–0.26% unprotonated nicotine (P.P, Madhu, BHR, and Double Black Royal Touch) while the products with basic pH (8.17–10.07) had unprotonated nicotine levels ranging from 58.55 to 99.12%. The Chaini Khaini with pH 9.33 had unprotonated nicotine of 95.33%. Gul sample had unprotonated nicotine in the range of 78.79%-99.12%. Jora panga Gul had a high level of unprotonated nicotine. The Pan Masala brands we tested did not contain any nicotine. The nicotine levels for the Coresta standards were 7.07–16.34 (Table 3).

Nine distinct flavors were detected in the tested SLT products (Table 4). The findings revealed that menthol was the most consistently present ingredient. Menthol concentrations in Zarda samples ranged from 4145.40 to 9681.42 µg/g, in chewing tobacco from 296.52 to 6617.37 µg/g, and in Pan Masala from 2371.62 to 5156.51 µg/g. The menthol content of the Khaini and Kharra samples was 5377.51 and 630.74 µg/g, respectively. Menthol has the ability to improve the delivery of nicotine. The amount of eucalyptol discovered in Zarda, Pan Masala, and Khaini was between 118.16 and 247.77 µg/g. Only Zarda and chewing tobacco had camphor, with concentrations of 148.67 and 219.32 µg/g, respectively. Coumarin was identified in a range of concentrations in Zarda, chewing tobacco, and Khaini, ranging from 112.33 to 244.25 µg/g. In one of the chewing tobacco samples, diphenyl ether was detected at a concentration of 10.89 µg/g. AR Chand and Jora Panja Gul had high nicotine levels without flavors, whereas Gulab Marka Gul contained coumarin 189.26 µg/g. We also looked for methyl salicylate, ethyl salicylate, cinnamaldehyde, and eugenol, but they were not detected.

**Table 4:** Concentration (μg/g, wet) of flavor -related compounds (n = 3) in Smokeless Tobacco Products.

Smokeless tobacco type	Name of the product	Eucalyptol	Camphor	Menthol	Diphenyl ether	Coumarin
Gudhaku	Tota chaap	–	–	–	–	–
Kharra	Golden babu	–	–	630.74	–	–
Khaini	Chaini khaini	244.59	–	5377.51	–	145.28
Gul	A R chand Gul	–	–	–	–	–
	Jora panja Gul	–	–	–	–	–
	Gulab marka Gul	–	–	–	–	189.26
Zarda	Np-01	165.81	148.67	9681.42	–	244.25
	Sir S zarda	–	–	4145.40	–	–
Chewing Tobacco	No brand-Loose tobacco	–	–	–	–	–
	P P tobacco	–	–	296.52	-	112.33
	Kuber tobacco	–	219.17	-	10.89	–
	Madhu chewing tobacco	–	–	2023.99	–	–
	BHR chewing tobacco	–	–	1826.44	–	164.26
Pan Masala	Double black royal touch	–	–	6617.37	–	–
	Kamala pasand pan masala	118.16	–	2371.62	–	–
	Pan parag premium	–	–	4554.58	–	–
	Rajnigandha pan masala	247.77	–	6513.97	–	–
	Vimal pan masala	178.96	–	5148.41	–	–
	Raj niwas premium	186.56	–	5156.96	–	–

## Discussion

**Figure 3:**
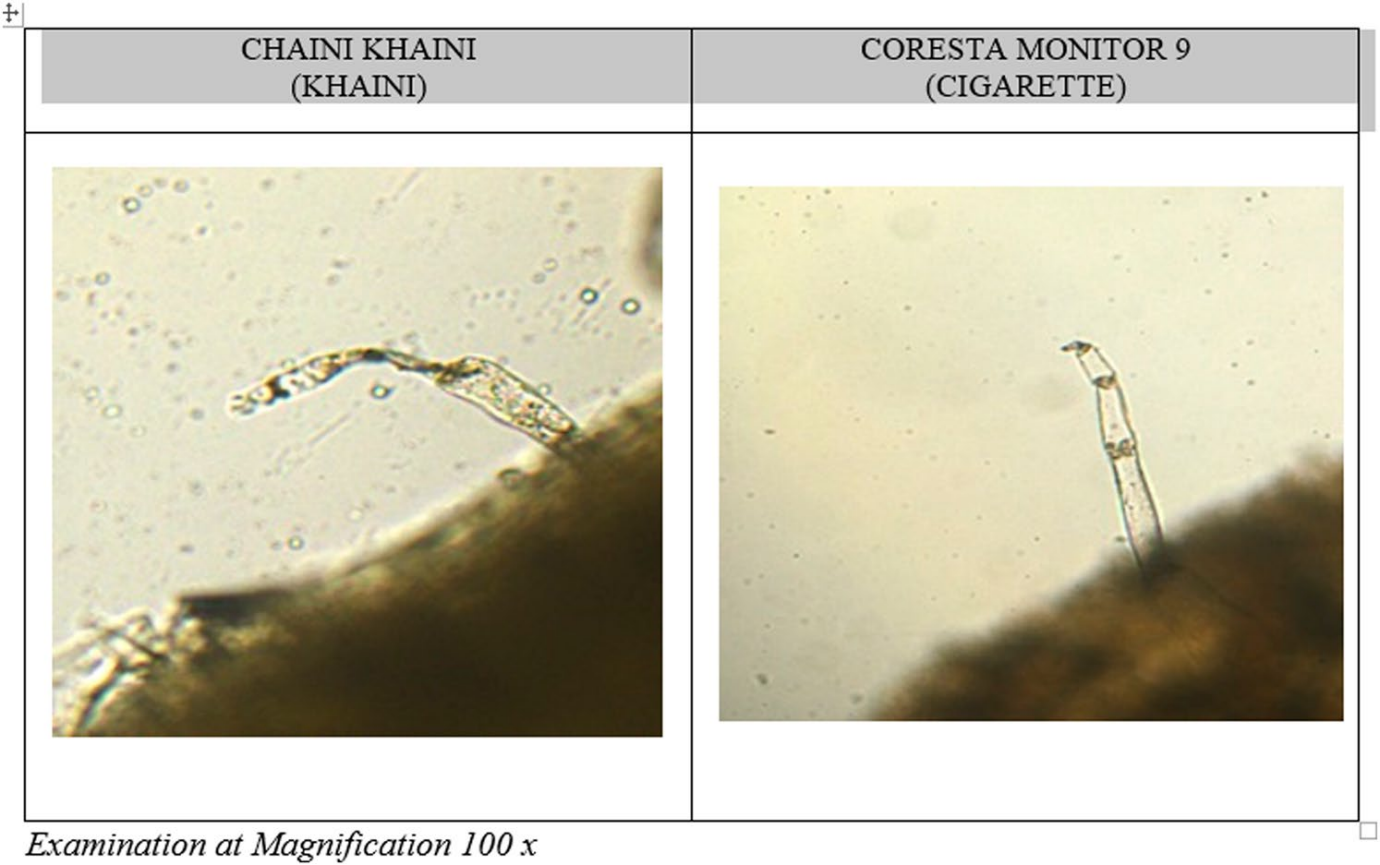
Cellular Microscopic Images showing trichomes in Khaini Ref Product and CM 9 Cigarette.

The current research study thoroughly examined pH, moisture, nicotine (protonated and unprotonated), and flavors in SLT available in India. It was found that the majority of commonly used SLT products had high levels of un-protonated nicotine and a basic pH state. Microscopic examination indicated that the Chaini Khaini was a finely ground tobacco product made with pasteurized air- or sun-cured tobacco that comes in tiny teabag-like sachets, while other Khaini brands were not finely processed. SLT had tobacco as reference material, therefore the photos were illustrative regardless of brand and type. The pH of SLT products is critical in determining the amount of un-protonated (or “free base”) nicotine, which impacts nicotine bioavailability^32^. Nicotine is found in both protonated (charged) and un-protonated (uncharged) forms in tobacco and tobacco smoke. Un-protonated nicotine is rapidly absorbed in the mouth, and the rate of absorption is a primary factor of addiction for nicotine, as it is for other substances^33,34^. Using an alkalinizing agent, such as the addition of ammonium bicarbonate, to change the pH level of the product and increase the amount of un-protonated nicotine is one way to manage nicotine delivery^35^ (Fig. 3).

Use of an ingredient that enhances salivation, like acetic acid, is another way to improve nicotine absorption of SLT products. Saliva production increases moistening of the tobacco held in the mouth (the plug) and facilitates nicotine extraction. Ammonium bicarbonate and acetic acid are used in the production of SLT.

The total and un-protonated nicotine levels of SLT products were consistent with our findings for Zarda, Gutkha, and Khaini in a prior investigation^29^. Gul, Kharra, and loose tobacco products, which are extensively consumed in India are also included in the current study. Gul, Kharra, and loose tobacco products included high levels of un-protonated nicotine, which delivered a considerable amount of nicotine to the user and were linked to health risks and addiction. Gulab Marka Gul with pH 9.63 had the greatest nicotine content (both total and un-protonated) of 31.25 mg/g. Similar Bangladeshi yields^36^ had mean nicotine levels of 31 mg/g, which were three times greater than SLT brands from Pakistan. (10 mg/g powder)^29,37^. Although nicotine was not detected in the Pan Masala samples, data from the National Tobacco Testing Labs (NTTL, India) (The Hindu, April 8, 2015) reveals that it is a leading cause of oral submucous fibrosis that often progresses to oral cancer^10,24^.

The nicotine, un-protonated nicotine, and flavour concentrations described in this paper are provided on a wet-weight basis. Nicotine has a bitter and disagreeable taste, thus flavouring is used to conceal it and make the product more palatable and appealing. Eucalyptol, camphor, methyl salicylate, ethyl salicylate, menthol, eugenol, cinnamaldehyde, coumarin, and diphenyl ether were among the nine regularly used flavourings. The additional flavour components in Zarda and Khaini were higher than the rest of the SLT products (Table 4). Stanfill et al.^27^ studied flavours in SLT products' and reported Menthol (range: 160–21,700 µg/g) was the most commonly used ingredient, followed by diphenyl ether (7.05–7.380 µg/g), coumarin (5.94–1,420 µg/g), eugenol (25.2–1250 µg/g), and camphor (6.94–1160 µg/g). Methyl salicylate (8.31–75.0 µg/g), pulegone (6.40–74.0 µg/g), and ethyl salicylate (10.5–16.0 µg/g) were among the compounds with lower amounts. According to our findings, menthol levels were 296.52–9681.42 µg/g, coumarin 112.33–244.25 µg/g, camphor (148.67–219.17 µg/g, eucalyptol 118.16–247.77 µg/g and diphenyl ether 10.89 µg/g was present in one product only. Khaini had high pH, nicotine, and un-protonated nicotine, but Zarda had very high total nicotine concentrations (21.9–32.9 mg/g). Menthol, camphor, coumarin eucalyptol, and diphenyl ether were the most commonly used flavors. Few SLT products had methyl salicylate, pulegone, or ethyl salicylate (18–20). We found no ethyl, methyl salicylate, cinnamaldehyde, or eugenol among the tested SLT products.

Despite the fact that SLT poses a serious health risk, little research has been done on the effects of oral exposure to these substances. Microscopic examination revealed that khaini samples went through fine processing. Customers may be drawn to these expertly packaged and refined products. Zarda has a lot of additives, which may be because it contains a variety of flavors, spices, and other ingredients, including tobacco and areca nut^29,31^. Even though SLTs are illegal in some Indian states and jurisdictions, the products are still sold and smuggled into the market because the bans'/regulations are not strictly enforced. In addition, SLT products like Gutkha and Pan Masala contain additional ingredients like areca nut, cardamom, and slaked lime, which increases the toxicity and addictiveness of the product. These products become more appealing because of certain flavoring ingredients' more seductive aromas and tastes, but they can also be toxic and dangerous. The effects of SLT product use on health need to be described through further study.

## Conclusion

Unlike cigarettes, the contents of SLT products are poorly researched and documented, especially in the South-East Asia Region, which has the highest prevalence of use of these products. Many countries with high prevalence of SLT use face regulatory challenges in the absence of testing facilities and relevant evidence regarding the contents, addiction potential, toxicity and health effects of these products. The microscopic examination in this study provided an added level of information to aid enforcement activities, in identifying SLTs, especially where such products are prohibited in some jurisdictions. The findings of this study would have important ramifications for regulating the use of SLT products as the evidence could drive and strengthen policies, legislations, extant bans as well as the implementation of WHO FCTC Articles to pave the way for effective tobacco control in the countries where SLT use is prevalent.

## Data Availability

The datasets generated during and/or analyzed during the current study are not publicly available due to confidential issues but are available from the corresponding author on reasonable request.

## Abbreviations

SLT: Smokeless tobacco
GCMS: Gas chromatograph, and mass spectrometer
FID: Flame ionization detector
SEAR: South-East Asia Region
SOP: Standard operating procedure
WHO TobLabNet: World Health Organization Tobacco Laboratory Network
WHO FCTC: World Health Organization Framework Convention on Tobacco Control
